# Clinical data analysis of 86 patients with invasive infections caused by *Malassezia furfur* from a tertiary medical center and 37 studies

**DOI:** 10.3389/fcimb.2023.1079535

**Published:** 2023-06-29

**Authors:** Xiaohui Zhang, Fei Jin, Fang Ni, Yuqiao Xu, Yanfei Lu, Wenying Xia

**Affiliations:** ^1^ Department of Laboratory Medicine, Jiangsu Province Hospital and Nanjing Medical University First Affiliated Hospital, Nanjing, China; ^2^ Branch of National Clinical Research Center for Laboratory Medicine, Nanjing, China

**Keywords:** *Malassezia furfur*, invasive infection, fungemia, pulmonary infection, premature infant, indwelling catheter, amphotericin B

## Abstract

**Objective:**

*Malassezia furfur* (*M. furfur*) is a lipophilic, conditionally pathogenic yeast that mainly causes skin infections, but the reports of related invasive infections are increasing. The aim of this study is to provide clinical data to assist physicians in the management of patients with invasive infections caused by *M. furfur*.

**Methods:**

A case of pulmonary infection caused by *M. furfur* in a hematopoietic stem cell transplant patient for aplastic anemia was reported. In addition, the literature on invasive infection by *M. furfur* published in PubMed and Web of Science in English until 31 July 2022 was reviewed.

**Results:**

Clinical data analysis of 86 patients (from 37 studies and our case) revealed that most of them were preterm (44.2%), followed by adults (31.4%). *M. furfur* fungemia occurred in 79.1% of the 86 patients, and 45 of them were clearly obtained from catheter blood. Other patients developed catheter-related infections, pneumonia, peripheral thromboembolism, endocarditis, meningitis, peritonitis and disseminated infections. Thirty-eight preterm infants had underlying diseases such as very low birth weight and/or multiple organ hypoplasia. The remaining patients had compromised immunity or severe gastrointestinal diseases. 97.7% of patients underwent invasive procedures and 80.2% received total parenteral nutrition (TPN). Fever, thrombocytopenia and leukocytosis accounted for 55.8%, 38.4% and 24.4% of patients with *M. furfur* invasive infections, respectively. 69.8% of the patients received antifungal therapy, mainly amphotericin B (AmB) or azoles. Of 84 patients with indwelling catheters, 58.3% underwent the removal of catheters. TPN were discontinued in 30 of 69 patients. The all-cause mortality of 86 patients was 27.9%.

**Conclusions:**

*M. furfur* can cause a variety of invasive infections. These patients mostly occur in premature infants, low immunity and severe gastrointestinal diseases. Indwelling catheters and TPN infusion are major risk factors. AmB, l-AmB and azoles are the most commonly used agents, and simultaneous removal of the catheter and termination of TPN infusion are important for the treatment of *M. furfur* invasive infections.

## Introduction

*Malassezia* are yeast species belonging to Basidiomycota, Ustilaginomycotina, and class Malasseziomycetes, which are commonly found on the skin surfaces of human body and other warm-blooded animals (Theelen et al., 2021). Currently, the *Malassezia* genus comprises 18 species with numerous functionally distinct strains based on morphology, microstructure, physiological biochemistry and molecular biology (Vijaya Chandra et al., 2021). As a conditionally pathogenic fungus, it is well known for causing a variety of skin diseases such as tinea versicolor, seborrheic dermatitis/dandruff, atopic dermatitis, folliculitis and psoriasis (Abdillah and Ranque, 2021). Besides these skin disorders, invasive infections caused by *Malassezia* have been increasingly reported in recent years.

*Malassezia furfur* (*M. furfur*) is the most encountered species from bloodstream infections in the *Malassezia* genus, followed by *M. pachydermatis* and *M. sympodialis*, especially in premature neonates and immunocompromised hosts (Rhimi et al., 2020). Apart from fungemia, pneumonia, peritonitis, meningitis and disseminated infection caused by *M. furfur* have been continuously reported worldwide in recent years (Shek et al., 1989; Johnson et al., 1996; Rosales et al., 2004; Baker et al., 2016). However, the epidemiology of these infections caused by *M. furfur* has not been well investigated. Due to its lipid-dependent nature, *M. furfur* infections are likely to be underestimated in routine clinical cultures (Theelen et al., 2018). The non-specific clinical manifestations of these infections make the diagnosis of *M. furfur* even more difficult.

Here, we describe a case of pulmonary infection caused by *M. furfur* in a patient undergoing hematopoietic stem cell transplantation (HSCT) for aplastic anemia (AA). As the largest tertiary medical center in the region, *M. furfur* is isolated from respiratory specimen for the first time in our hospital, which arises our great interest. In order to improve the understanding of *M. furfur* among clinicians and laboratory personnel, this article reviews the cases of invasive infections caused by it. In addition, the latest advances in the pathogenesis, diagnosis and treatment of *M. furfur* invasive infections were summarized and discussed.

## Materials and methods

### Case presentation

A 17-year-old male underwent HSCT in our hematology department 7 months ago for AA. Immunosuppressant and hormone therapy were routinely administered after transplantation. The patient developed fever 3 days before admission, and a chest computed tomography (CT) examination at the local hospital indicated severe infection of both lungs. At admission, laboratory tests revealed a normal white blood cell count (WBC) but with a markedly reduced platelet count (56×10^9^/L) and low hemoglobin (105 g/L). His C-reactive protein (CRP) was markedly elevated (52.1 mg/L), and the level of galactomannan was normal. Multi-row CT chest scan showed multiple patchy ground-glass shadows in the lower lobe of both lungs, considering the possibility of infectious lesions.

**Figure 1:**
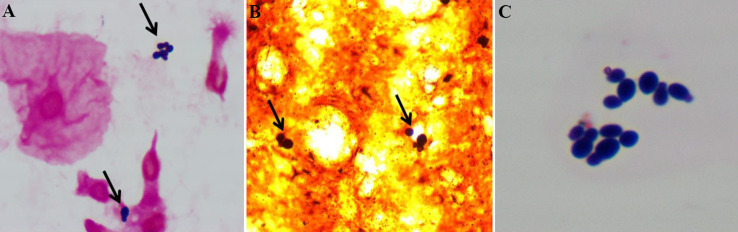
**(A)** After centrifugation of bronchoalveolar lavage fluid, gram staining of the precipitate revealed gram-positive and ‘bottle-shaped’ budding yeast cells (black arrows indicated). **(B)** Bronchoalveolar lavage fluid staining with gomori methenamine silver showed dark brown and budding spores (black arrows indicated). **(C)** Gram staining of the colonies showed ‘bottle-shaped’ budding yeast cells, with a collarette-like thickening seen at the junction between the budding and the mother spores.

**Figure 2:**
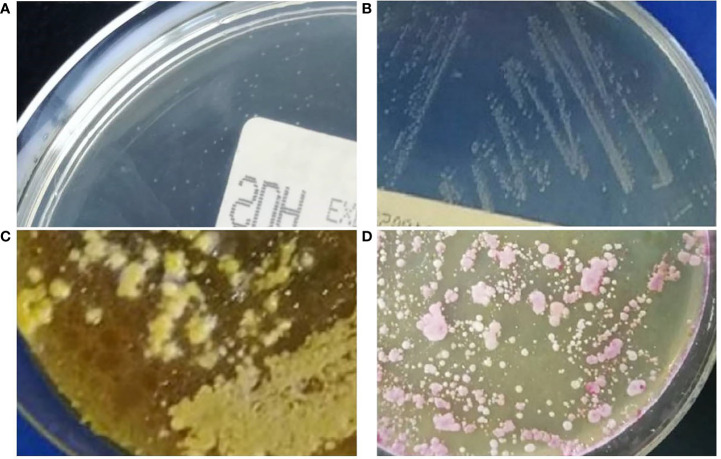
**(A)** Dust-like colonies were found in bronchoalveolar lavage fluid cultured on sabouraud dextrose agar plate for 3 days at 35°C. **(B)** Small creamy and fragile colonies were observed on the sabouraud dextrose agar plate after 5 days at 35°C. **(C)** After 3 days of incubation on sabouraud dextrose agar covered with sterile olive oil at 35°C, the morphology of colonies was cheesy, raised, smooth or rough. **(D)** The colonies appeared pink on CHROMagar™ Candida with sterile olive oil supplementation after 3 days at 35°C.

Blood cultures drawn from peripheral vein and central venous catheter (CVC) were negative. No abnormality was observed in sputum culture. Due to the aggravation of infection, bronchoscopy with bronchoalveolar lavage was performed. After centrifugation of bronchoalveolar lavage fluid (BALF), gram staining and Gomori methenamine silver (GMS) staining revealed some bottle-shaped and budding yeast cells (**Figures 1A, B**). The BALF was cultured to sabouraud dextrose agar (SDA) and columbia blood agar medium and incubated at 35°C. Dust-like colonies grew on the SDA after 3 days of incubation (**Figure 2A**) but not on blood agar. After 5 days of culture, small creamy and fragile colonies were observed on SDA plate (**Figure 2B**). Gram staining of the colonies showed a culster of budding yeast cells, with a collarette-like structure seen at the junction between the budding and the mother spore (**Figure 1C**). The colonies were transferred to another SDA and CHROMagar™ Candida covered with a thin layer of sterile olive oil for subculturing. After 3 days of incubation, the morphology of colonies on the SDA covered with olive oil (**Figure 2C**) were cheese-colored, raised, smooth or rough, and pink on CHROMagar™ Candida covered with olive oil (**Figure 2D**). The colonies were identified as *M. furfur* by Vitek 2 Compact microbial identification system (BioMérieux) and matrix-assisted laser desorption ionization time-of-flight mass spectrometry (MALDI-TOF MS) with a 99.9% confidence level. Furthermore, the second-generation sequencing results of BALF showed that the number of sequences of *M. furfur* was as high as 5830.

Originally, *pneumocystis carinii* infection was highly suspected clinically, given the patient’s condition. Biapenem, sulfamethoxazole-trimethoprim and caspofungin were administered as anti-microbial therapy. When the pathogen was confirmed as *M. furfur*, caspofungin was replaced with voriconazole (VOR, 200mg every 12 hours) for antifungal treatment. After 14 days of treatment, the patient had no fever, and the symptoms of chest distress and asthma were improved. The results of chest CT reexamination showed that the infection was better than before. The patient was transferred from the intensive care unit to the hematology department and continued oral VOR (200mg twice daily) for 14 days. At follow-up 3 months later, the patient continued to show clinical improvement with no signs of infection.

### Literature review

We conducted literature searches in PubMed and Web of Science, and the literature was published until 31 July 2022. The query was ‘*Malassezia furfur*’, and article type choosed ‘Case report’. All relevant, available full texts published in English were extracted and the details of clinical data were retrieved.

## Results

**Figure 3:**
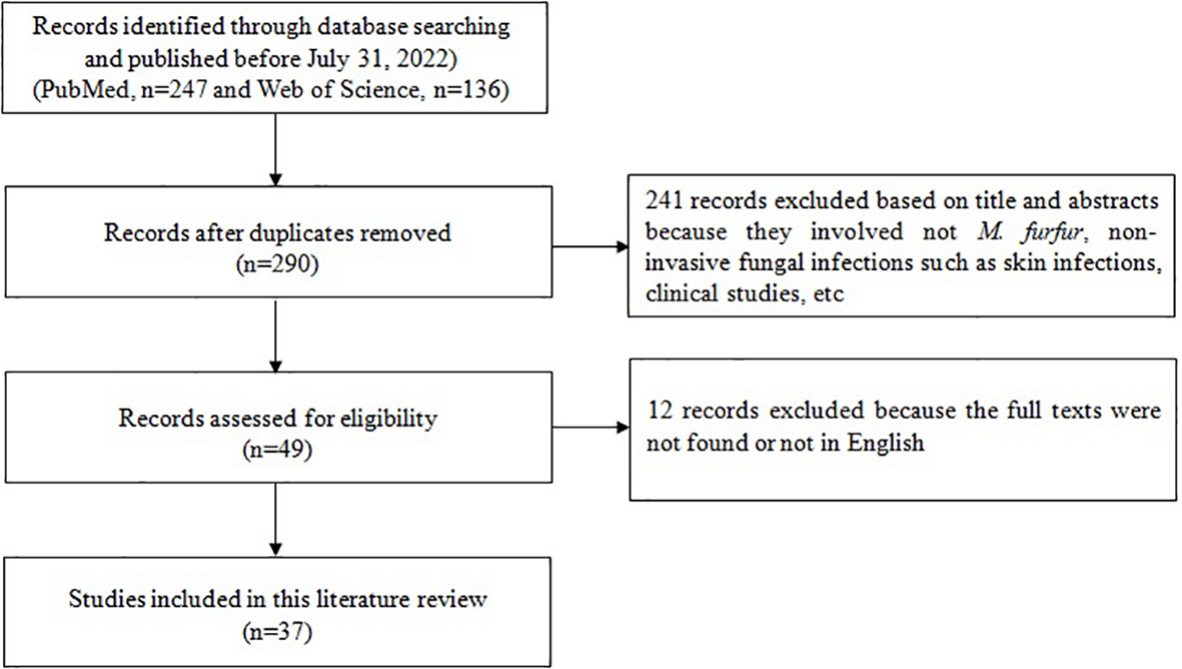
Flow-chart of articles related to case reports of invasive infection caused by *Malassezia furfur*.

A total of 37 articles including 85 patients were obtained through retrieval (**Figure 3**) and included in the data analysis together with the case of this study. The clinical data of 86 patients with *M. furfur* invasive infections were shown in **Table 1**. The geographic distribution, demographic characteristics, site of infection, underlying disease, clinical feature, laboratory data, treatment and patient outcome were summarized and analyzed.

**Table 1:** Clinical data of 86 patients with *M. furfur* invasive infections.

No.	Gender/Age (yr)	Culture specimen type	Caused disease	Use TPN/catheter	Underlying disease	Laboratory data	Treatment	Prognosis	Author, year
1	M/17	BALF	Pulmonary infection	no/yes	AA, after HSCT	Thrombocytopenia, elevated CRP	VOR	Survived	This study
2	M/3	Blood drawn from CVC, catheter tip	Fungemia	no/yes	High-risk neuroblastoma	Neutropenia	CVC removal	Survived	(Tetsuka et al., 2022)
3	M/33	Blood, valve vegetation	Endocarditis	no/no	After resection of back melanoma, CHD	Low hemoglobin	VOR, l-AmB, valve replacement	Survived	(Granok, 2020)
4	M/28 + 6wk*	Blood	Fungemia	yes/yes	VLBW	Thrombocytopenia	AmB	Survived	(Chen et al., 2020)
5	F/2	Blood, CVC	Fungemia	no/yes	Spine osteomyelitis	NA	l-AmB, oral FLZ, CVC removal	Survived	(Pedrosa et al., 2018)
6	F/68				HIV, SIRS, oral epidermoid carcinoma	NA	l-AmB, CVC removal	Died	
7	M/60				Hodgkin’s disease, ARDS, HIV	NA	l-AmB, CVC removal	Died	
8	F/12	BALF, blood	Pneumonia, fungemia	yes/yes	Williams syndrome, oral and gastrojejunal feeding intolerance, hypoxemia, cyclic vomiting	Leukocytosis, elevation in ESR, CRP and IgE	l-AmB, oral FLZ, CVC removal	Survived	(Baker et al., 2016)
9	F/0.25	Blood	Fungemia	yes/yes	Gastroschisis, ileostomy surgery	Leukocytosis, elevated CRP	l-AmB, TPN was discontinued	Survived	(Oliveri et al., 2011)
10	F/12	Sputum	Pneumonia	yes/yes	MLD, after SCT	NA	Micafungin	Died	(Blaes et al., 2009)
11	M/68	BALF			CLL/MDS, after SCT, GVHD	NA	Intravenous l-AmB, AmB nasal irrigations	Died	
12	M/42				AML, after SCT	Neutropenia	VOR, caspofungin, AmB, pneumonectomy	Died	
13	F/61				MDS, after SCT	NA	VOR, AmB	Survived	
14	M/53	Blood	Fungemia	yes/yes	Crohn’s disease, short-bowel syndrome	Elevated liver enzyme levels	AmB, daptomycin, vancomycin	Survived	(Bhargava and Longhi, 2007)
15	M/23wk*	Blood, catheter tip, CSF	Fungemia, meningitis	yes/yes	VLBW, chronic lung disease, NEC, intraventricular hemorrhage	Thrombocytopenia, elevated CRP	AmB, catheter removal	Died	(Rosales et al., 2004)
16	F/34wk*	Blood drawn from CVC	Fungemia, peripheral thromboembolism	yes/yes	Intestinal perforation, after bowel resection and ileostomy	Leukopenia, thrombocytopenia and elevated CRP	AmB, CVC removal, TPN was reduced	Survived	(Kessler et al., 2002)
17	M/39	Blood	Fungemia	no/yes	Industrial accident, spinal shock, respiratory failure, after mechanical ventilation	Blood counts were normal	l-AmB, oral FLZ, CVC removal	Survived	(Chu and Lai, 2002)
18	M/30wk*	Catheter	Catheter-related infection	yes/yes	VLBW, after surgery for ileum perforation	Blood counts were normal	Urokinase, catheter removal	Survived	(Nguyen et al., 2001)
19	F/25wk*				VLBW, chronic lung disease, RDS	Thrombocytopenia, leukopenia	AmB, recombinant tissue plasminogen activator, TPN was discontinued	Survived	
20	F/45	Blood, catheter tip	Fungemia	yes/yes	Crohn’s disease, after multiple abdominal surgeries	Leukopenia, thrombocytopenia	AmB, CVC removal, TPN was discontinued	Survived	(Schleman et al., 2000)
21	M/57	Dialysis fluid	CAPD Peritonitis	NA/yes	DM, end-stage renal failure, CAPD	CAPD fluid revealed the elevation in WBC	Antifungal therapy, catheter removal, peritoneal cavity was washed out	Survived	(Johnson et al., 1996)
22	M/10wk*	Blood drawn from catheter, catheter tip	Fungemia	yes/yes	Hirschsprung disease, multiple bowel syndrome	Leukocytosis, low hemoglobin	AmB, catheter removal	Survived	(Glaser and Atwater, 1995)
23	M/30wk*	Central line	Solitary pyogenic liver abscess	yes/yes	VLBW, NEC, ileal resection	Leukocytosis, thrombocytopenia	AmB, central line removal	Died	(Doerr et al., 1994)
24	M/30wk*	CVC	CVC occluded, and adhered to the vein wall or surrounding tissue	yes/yes	VLBW, RDS, pneumothorax	Leukocytosis, thrombocytopenia	CVC removal	Survived	(Kim et al., 1993)
25	M/25wk*				VLBW, RDS, NEC, intestinal perforation	Thrombocytopenia	CVC removal	Survived	
26	M/0.25	Blood drawn from CVAD	Fungemia	no/yes	Severe combined immunodeficlency disease	Neutropenia, thrombocytopenia	AmB through CVAD	Survived	(Barber et al., 1993)
27	M/4				ALL, chemotherapy	Leukocytosis	AmB through CVAD	Survived	
28	F/24				Osteogenic sarcoma, chemoradiotherapy	Neutropenia, thrombocytopenia	AmB through CVAD	Survived	
29	F/42				CLL, chemoradiotherapy	Leukocytosis, neutropenia, thrombocytopenia	AmB through CVAD	Died	
30	M/28			yes/yes	Embryonal rhabdomyosarcoma, radiation therapy	Blood counts were normal	AmB through CVAD	Survived	
31	M/40				AA, chemoradiotherapy	Neutropenia, thrombocytopenia	AmB through CVAD	Died	
32	F/2			no/yes	Medulloblastoma, chemotherapy	Blood counts were normal	AmB through CVAD, CVAD removal due to persistant *M. furfur* infection	Survived	
33	M/40	Blood, catheter tip	Fungemia	no/yes	DM, broad-spectrum antibiotics, hemodialysis	NA	Catheter removal	Survived	(Myers et al., 1992)
34	M/66		Fungemia		Lung cancer, broad-spectrum antibiotics	NA	AmB, miconazole	Died	
35	F/7	Blood	Fungemia	yes/yes	ALL, chemotherapy, methyl prednisolone, broad spectrum antibiotics	NA	AmB, 5-fluorocytosine	Died	(Masure et al., 1991)
36	M/35	Blood drawn from CVC	Fungemia	yes/yes	Bowel resection for a large intraperitoneal injury	NA	AmB administered through CVC, TPN was discontinued	Survived	(Arnow and Kushner, 1991)
37	M/28wk*	Blood drawn from CVC	Fungemia	yes/yes	VLBW, tyrosinosis and Rossier’s syndrome	Leukocytosis, thrombocytopenia	Miconazole, TPN was discontinued	Survived	(Surmont et al., 1989)
38	F/31wk*				VLBW, BPD	Blood counts were normal	TPN was discontinued, catheter removal	Survived	
39	M/33wk*				2200g birth weigh, hyaline membrane disease, PDA and BPD	Blood counts were normal	Miconazole, TPN was discontinued	Survived	
40	M/28wk*				VLBW, BPD, Meckel’s diverticle, ileum resection	Leukocytosis	Miconazole, TPN was discontinued	Died	
41	M/33wk*	Blood, CVC tip			1800g birth weigh, hyaline membrane disease evolving to BPD	NA	Cloxacillin, netilmicin, miconazole, catheter removal	Survived	
42	F/29wk*	Blood			VLBW, hyaline membrane disease resulting in BPD	NA	Miconazole, CVC removal, TPN was discontinued	Survived	
43	M/25wk*	Autopsy	Disseminated Infection	yes/yes	VLBW, PDA	Leukocytosis, thrombocytopenia	A variety of antibiotics	Died	(Shek et al., 1989)
44	F/26wk*				VLBW, intubation, PDA, NEC, a segment of terminal ileum was resected	NA	A variety of antibiotics	Died	
45	M/25wk*				VLBW, intubation, NEC, pneumatosis intestinalis, PDA, BPD	Leukocytosis	Antibiotics	Died	
46	M/22	Peritoneal dialysis fluid	Peritonitis	NA/yes	End-stage renal failure, peritoneal dialysis	NA	Ketoconazole, FLZ, catheter removal	Survived	(Gidding et al., 1989)
47	M/70	Blood, hip aspirate	Fungemia	no/no	Hairy cell leukemia, prostate adenocarcinoma, splenectomy, tinea versicolor (have been cured)	Serum triglycerides were elevated	AmB, oral cephalexin and ketoconazole	Survived	(Wurtz and Knospe, 1988)
48	F/0.5	Blood drawn from CVC and the catheter	Fungemia	yes/yes	Gastroschesis, postoperative short gut syndrome	NA	Miconazole and AmB through the catheter, catheter removal, TPN was discontinued	Survived	(Powell and Marcon, 1987)
49	M/0.42				Short gut syndrome secondary to gastroschesis and jejunal atresia	NA	Miconazole through the catheter, catheter removal due to persistant infection, TPN was discontinued	Survived	
50	M/46	Blood, CVC	Fungemia	yes/yes	ANLL, Crohn’s disease, hemicolectomy, ileostomy, chemotherapy, broad spectrum antibiotics	Thrombocytopenia	Hickman catheter removal, TPN was discontinued	Survived	(Middleton and Lowenthal, 1987)
51	M/62	Blood drawn from CVC	Fungemia	yes/yes	Small bowel infarction and partial small bowel resection, pneumonectomy for carcinoma	Leukocytosis	AMB	Died	(Garcia et al., 1987)
52	M/49				Short bowel syndrome, seizures, recurrent aspiration pneumonia	Serum triglycerides were elevated	AmB through CVC, TPN was discontinued	Died	
53	F/25-26wk*	Blood drawn from catheter	Fungemia	yes/yes	VLBW, hyperbilirubinemia, surgical ligation for PDA, NEC, multiple courses of antibiotics	Thrombocytopenia	AmB, CVC removal, TPN was discontinued	Survived	(Dankner et al., 1987)
54	M/32wk*				1520g birth weigh, PDA, NEC and apnea of prematurity	NA	AmB, CVC removal	Survived	
55	M/26wk*				VLBW, chronic lung disease and recurrent apnea, NEC	Leukopenia, thrombocytopenia	Umbilical catheter removal, TPN was discontinued	Survived	
56	M/38wk*				Persistent fetal circulation, hydrocephalus, and congenital malformations	Blood counts were normal	AmB through the catheter, TPN was discontinued, catheter removal due to persistant infection	Died	
57	M/29wk*				VLBW, NEC and bowel perforation, partial colectomy	NA	Catheter removal, TPN was discontinued	Survived	
58	M/71				Adenocarcinoma, surgery	Blood counts were normal	TPN was discontinued	Survived	
59	M/57				Subtotal colectomy, surgeries for hemorrhoids and anal fissures, aortic valve replacement	NA	Catheter removal	Survived	
60	F/34	Blood drawn from catheter	Fungemia	yes/yes	Cervical cancer, radiation therapy, ileostomy and drainage of a pelvic abscess	NA	AmB	Died	(Brooks and Brown, 1987)
61	NA/25wk*	Blood drawn from CVC	Fungemia	yes/yes	VLBW, BPD, PDA	Blood counts were normal	Catheter removal	Survived	(Alpert et al., 1987)
62	NA/27wk*				VLBW, BPD	Thrombocytopenia	Catheter removal	Survived	
63	NA/37wk*				2910g birth weigh, CHD, partial bowel resection	Blood counts were normal	Catheter removal	Died	
64	NA/27wk*				VLBW, NEC	Leukocytosis, thrombocytopenia	AmB, 5-fluorocytocine, catheter removal	Died	
65	NA/31wk*				VLBW, NEC	Leukopenia, thrombocytopenia	Catheter removal	Survived	
66	NA/40wk*				3400g birth weigh, NEC	Thrombocytopenia	Catheter removal	Survived	
67	NA/28wk*				VLBW, NEC	Leukopenia, thrombocytopenia	AmB, catheter removal	Survived	
68	F/54days^#^	Blood, CVC tip	Fungemia	yes/yes	VLBW, BPD, CHF, HLD, hypothyroidism	Leukocytosis, thrombocytopenia	AmB, 5-fluorocytosine, TPN was discontinued	Died	(Redline et al., 1985)
69	F/76days^#^	Autopsy lung tissue	Severe pneumonia		VLBW, BPD, CHF, HLD, hypothyroidism	Leukopenia, thrombocytopenia	TPN was discontinued	Died	
70	F/1.5^#^	Blood	Fungemia		Hirschsprung’s disease, colostomy	Thrombocytopenia	TPN was discontinued	Survived	
71	M/16	Blood	Fungemia		Crohn’s disease, total small bowel resection, immunosuppressive therapy	Leukopenia	TPN was discontinued	Survived	
72	M/22	Blood, open lung biopsy	Systemic infection		AA, BMT, severe gastrointestinal GVHD, immunosuppressive therapy	Granulocytopenia, thrombocytopenia	AmB, TPN was discontinued	Survived	
73	M/48	Blood, lung Biopsy	Systemic infection		Hemorrhagic pancreatitis with multiple enterocutaneous fistulae, alcohol abuse	Blood counts were normal	TPN was discontinued	Survived	
74	F/34wk*	Blood drawn from CVC	Fungemia	yes/yes	1500g birth weigh, cardiac malformations, after surgical for NEC	Thrombocytopenia	AmB, catheter removal	Survived	(Long and Keyserling, 1985)
75	M/36wk*				2500g birth weigh, bowel volvulus, surgery	Leukocytosis	Catheter removal	Survived	
76	F/27wk*				VLBW, respiratory failure, gastric perforation	Neutropenia, thrombocytopenia	AmB, catheter removal, TPN was discontinued	Survived	
77	F/30wk*				VLBW, resection of much of the small bowel for NEC	WBC count was 5,000/μl	AmB, catheter removal, TPN was discontinued	Survived	
78	M/32wk*	Blood drawn from CVC	Fungemia	yes/yes	1520g birth weigh	NA	AmB, catheter removal	Survived	(Dankner and Spector, 1985)
79	F/26wk*				VLBW, NEC, exchange transfusion for hyperbilirubinemia and ligation of PDA	NA	AmB, catheter removal	Survived	
80	M/33wk*	Blood drawn from CVC	Fungemia	yes/yes	VLBW, NEC, apnea of premature	Thrombocytopenia, neutrophilia	Catheter removal	Survived	(Powell et al., 1984)
81	F/27wk*				VLBW, BPD, seizures	Thrombocytopenia, neutrophilia	Catheter removal	Survived	
82	F/31wk*				VLBW, BPD, apnea of premature	Thrombocytopenia, neutrophilia	Catheter removal	Survived	
83	M/28wk*				VLBW, possible NEC, PDA	Thrombocytopenia	Catheter removal	Survived	
84	F/40wk*				Achalasia, aspiration pneumonia	Neutrophilia	Catheter removal	Survived	
85	M/3.5	Clot attached to CVC	Pulmonary infection and infarction	yes/yes	Short bowel syndrome after surgery for volvulus and ileal atresia	Leukocytosis	AmB, catheter removal, TPN was discontinued	Survived	(Hassall et al., 1983)
86	F/28wk*	Open-lung biopsy, autopsy	Pulmonary vasculitis	yes/yes	VLBW, RDS, BPD	Leukocytosis, neutrophilia	AmB and flucytosine	Died	(Redline and Dahms, 1981)

*gestational age, ^#^patient’s age at diagnosis.

M, male; F, female; NA, not available; BALF, bronchoalveolar lavage fluid; AA, aplastic anemia; HSCT, hematopoietic stem cell transplant; CRP, C-reactive protein; VOR, voriconazole; CVC, central venous catheter; CHD, congenital heart disease; l-AmB, liposomal amphotericin B; FLZ, fluconazole; SIRS, systemic inflammatory response syndrome; ARDS, acute respiratory distress syndrome; ESR, erythrocyte sedimentation rate; TPN, total parenteral nutrition; MLD, metachromic leukodystrophy; CLL, chronic lymphocytic leukemia; MDS, myelodysplastic syndrome; GVHD, graft-versus-host disease; AML, acutemyeloid leukemia; VLBW, very low birth weight; NEC, necrotizing enterocolitis; RDS, respiratory distress syndrome; DM, diabetes mellitus; CAPD, continuous ambulatory peritoneal dialysis; WBC, white blood cell count; CVAD, central venous access device; ALL, acute lymphocytic leukemia; BPD, bronchopulmonary dyplasia; PDA, patent ductus arteriosus; ANLL, acute non-lymphocytic leukemia; CHF, congestive heart failure; HLD, hyperalimentation liver disease; BMT, allogeneic bone marrow transplant.

The majority of 86 patients in this study were reported from USA with 68 patients (80%), 6 from Belgium, 3 each from China and Portugal, 2 in Australia, and 1 each from France, UK, Italy and Japan. 79.1% of the patients were isolated from the blood culture, of which 45 were definitely drawn from catheter blood. In addition, mycotic thrombosis around the indwelling catheter tip or leading to catheter occlusion, peripheral thromboembolism, pneumonia, endocardial neoplasms, meningitis, peritonitis, pulmonary vasculitis, inflammation of alveolar, bronchial and bronchiolitis, and disseminated infections (spread to heart, kidney, liver, spleen and pancreas) caused by *M. furfur* were reported.

62.0% were male in 79 patients with known gender. The majority of 86 patients were premature neonates (44.2%), followed by adults older than 18 years (31.4%) and infants younger than 1 year (12.8%). Among 38 premature infants, 78.9% were less than 32 weeks of gestational age and all had very low birth weight (VLBW, birth weight was less than 1500g). These premature infants were born with multiple organ hypoplasia. In addition, 12 patients had hematological malignancy and half of which underwent stem cell transplantation (SCT), and 11 patients had solid malignant tumor. Other patients reported in the literature presented with a variety of underlying diseases such as Crohn’s disease, Hirschsprung disease, short bowel syndrome, bowel surgery, and HIV infection.

97.7% of patients underwent invasive procedures, and predominantly with central venous access devices (CVADs). All 38 premature infants had CVADs and received total parenteral nutrition (TPN). Among the 48 patients of non-premature infants, 31 patients (64.6%) received intralipid emulsions, and 20 of them were statistically found to be caused by feeding intolerance, gastrointestinal disease and/or intestinal resection. The most common presentations were fever (55.8%), bradycardia (15.1%), apnea (14.0%), and respiratory distress (8.1%). Laboratory examination showed thrombocytopenia in 38.4% patients and leukocytosis in 24.4% patients. Leukopenia was found in 15 patients, and further analysis revealed that 6 of them were premature, and the other 9 had immunodeficiency disease or had received chemoradiotherapy or SCT for malignancy.

69.8% of the patients received anti-microbial therapy. Of 84 patients with indwelling catheters, 58.3% underwent the removal of catheters. Parenteral lipid emulsions were discontinued in 30 of 69 patients. Amphotericin B (AmB) or liposomal-AmB (l-AmB) was the most commonly used antifungal agent, accounting for 76.7% (46/60). 17 patients were treated with azole drugs, most of which were combined with AmB or sequential therapy. Of the 26 patients who did not receive antifungal therapy, 80.8% had catheters removed (partially simultaneous termination of TPN) and 34.6% terminated TPN. The all-cause mortality of 86 patients was 27.9%. Among the 24 patients who died, 15 cases of bacteremia, 5 of pneumonia and 4 of disseminated infection were caused by *M. furfur*. All deaths had CVADs, and 20 patients received TPN infusion. There were 22 deaths that were treated with antifungal therapy, 17 of which were treated with AmB or l-AmB. Only 6 of these 22 patients had the catheter removed.

## Discussion

The overall incidence of invasive fungal infection (IFI) was approximately 6 cases per 100 000 persons per year, with a mortality of 27.6% (von Lilienfeld-Toal et al., 2019). Recent studies have reported higher morbidity and mortality in neonates and immunocompromised patients, especially those with cancer and stem cell or solid-organ transplant (Bassetti et al., 2021; Puerta-Alcalde and Garcia-Vidal, 2021; Weimer et al., 2022). *Candida* and *Aspergillus* were generally considered to be the most common pathogens of invasive fungal disease (IFD). However, increasingly emerging opportunistic fungal (such as *Malassezia*) infections were being recognized and might play an important role in IFD (Miceli et al., 2011). The majority of invasive infections caused by *Malassezia* genus reported in recent years were associated with the lipid-dependent *M. furfur*. A 12-month study of fungal bloodstream infections in critical care patients showed a higher prevalence of *M. furfur* (2.1%) than *candida* spp. (1.4%) in patients with very complex underlying diseases or premature infants (Iatta et al., 2014). The severity of invasive infection caused by *M. furfur* could range from localized organ infection to fulminant fungemia with death (Devlin, 2006). Due to improper diagnosis, invasive infections caused by *M. furfur* were likely to be underestimated. In this study, we provided an overview of the epidemiology, risk factors, pathogenesis, clinical feature, diagnosis, treatment and clinical outcome of *M. furfur* invasive infections.

Several retrospective studies found that host immunosuppression played a dominant role in the development of IFI (Jenks et al., 2020). Congenital immune deficiency, prolonged neonatal intensive care units (NICU) stay, multiple courses of antibiotics and surgical treatment might increase the risk of *M. furfur* infection in premature infants. *M. furfur* colonization has been reported to occur in rates of 36.8% to 64% of infants hospitalized in NICU (Nguyen et al., 2001). Furthermore, there has been a nosocomial outbreak of systemic *M. furfur* infection in neonatal nurseries, with evidence of human-to-human transmission (Devlin, 2006). The presence and duration (>10 days) of granulocytopenia, treatment with glucocorticoids, chemoradiotherapy, and exposure to broad-spectrum antibiotics that caused changes in endogenous microbiota might be predisposing conditions for *M. furfur* infection. Limon et al. found that *Malassezia* was strongly associated with the inflammatory bowel disease, especially Crohn’s disease (Limon et al., 2019). In addition, *Malassezia* probably colonized internal organs in immunocompromised HIV patients (Abdillah and Ranque, 2021). The correlation of *M. furfur* infection with these underlying diseases deserved further investigation.

The risk of opportunistic fungal infection was increased by invasive procedures such as deep venous catheterization, mechanical ventilation and percutaneous drainage that breached the skin barrier and served as a port of entry and a nidus for pathogen infection (Jenks et al., 2020). Biofilms formation of *M. furfur* played an important role in its pathogenesis. It underwent multistep growth processes, including contacting with suitable substrates, adhesion to the biotic or abiotic surface by reversible hydrophobic interactions, production of polysaccharide material, biofilms maturation and dissemination (Inigo and Del Pozo, 2018). The correlation between hydrophobicity, adherence and biofilms formation in *M. furfur* species was examined and confirmed, as reported by other authors for *Candida albicans* (Borghi et al., 2011; Vavala et al., 2013). Human tissues, indwelling medical devices, other implanted foreign bodies and mucosal surfaces provided the substrate for biofilms colonization and infection (Boisvert et al., 2016). Once adhered to the surfaces, pathogens were extremely difficult to be eradicated, leading to a large number of nosocomial infections. The increased use of medical devices such as catheters and prosthetics contributed to the recurrent invasive infections and increased mortality rates among patients (Lagree and Mitchell, 2017).

*Malassezia* yeasts could produce a variety of enzymes, such as lipases and phospholipases that utilized lipids and proteases that utilized proteins (Vijaya Chandra et al., 2021). These enzymes might play complex roles in the human hosts, on one hand were involved in the pathogenesis, and on the other hand had a protective function (Rhimi et al., 2020). Studies found that *M. furfur* strains causing fungemia showed very high lipolytic enzyme activity, which were considered as virulence factors since they might be related to the colonization, proliferation, invasion and persistence in host tissues (Petrokilidou et al., 2019). However, *Malassezia* yeasts lacked fatty acid synthase and δ-9 desaturase, making them unable to synthesize lipids thus suggesting that they might need to inhabit lipid-rich skin or use exogenous lipids (Vijaya Chandra et al., 2021). Colonization of *M. furfur* on the surfaces of skin and catheter might increase the chance of invasive infections, and parenteral lipid emulsions could play an important role in their growth. Contamination during catheter placement, injection of contaminated lipids, hematogenous seeding of the catheter from a distant source, migration of *M. furfur* from the exit site on skin along the outer wall of the catheter and from the catheter hub through the lumen have been suggested as possible infection pathways (Marcon and Powell, 1987).

This study showed that invasive infections caused by *M. furfur* were special in that most of them occurred in prematurity, immunocompromised hosts, and patients requiring TPN via CVADs due to severe gastrointestinal disease and/or bowel resection. Given that these risk factors were largely unmodifiable, *M. furfur* invasive infections were likely to continue in the future. Studies have found that there were no differences in clinical features or laboratory markers between *candida* and *Malassezia* fungemia (Iatta et al., 2014). However, *M. furfur* fungemia appeared earlier and lasted longer than candidemia, which undoubtedly increased its mortality (Rhimi et al., 2020). In addition, infections caused by *M. furfur* seemed to be more complicated and more likely to lead to disseminated infections of the lungs, heart, kidneys, liver, spleen, and brain than *M. pachyderm* as another *Malassezia* causing fungemia (Huang et al., 2020). Hence, early diagnosis and timely and effective treatment were the key to reduce the mortality of invasive infections caused by *M. furfur*.

Isolation and accurate identification of *M. furfur* from the site of infection is currently the gold standard for the diagnosis of related infections. Unlike other studies, *M. furfur* grew on SDA plates in our case, which might differ in nutrient composition from plates from different manufacturers, or the small amount of lipids remaining in the specimen might have contributed to its growth. However, the dust-like colonies were easily overlooked, and its morphological characteristics were not typical due to poor growth. *M. furfur* did not grow well on SDA plate, but colony morphology could be clearly observed after cultured on SDA covered with sterile olive oil. Olive oil was composed of oleic acid and triglyceride esters of palmitic acid, which were good for promoting the growth of *M. furfur* (Devlin, 2006). It grew at temperatures between 25°C and 37°C, with optimal growth at 35°C-37°C (Dankner et al., 1987). If *M. furfur* infection was suspected, the clinician should alert the microbiology personnel at the time of culture submission. In our case, pink colonies of *M. furfur* were clearly visible on our modified chromogenic *Candida* medium (covered with sterile olive oil). MALDI-TOF MS was increasingly used and provided faster and more reliable identification at the *Malassezia* species level (Rhimi et al., 2021). In recent years, the development of molecular methods such as next-generation sequencing (NGS) had made it possible to identify yeasts directly from clinical specimens without the need to cultivate. Moreover, DNA sequencing and polymerase chain reaction-restriction fragment length polymorphism (PCR-RFLP) could differentiate the vast majority of *Malassezia* species (Didehdar et al., 2014; Lian et al., 2014).

Thus far, the antifungal susceptibility testing recommended by the Clinical and Laboratory Standards Institute (CLSI) and European Committee on Antimicrobial Susceptibility Testing (EUCAST) was not suitable for *Malassezia* species due to their lipid-dependent nature, slow growth and a tendency to form cell clusters (Rhimi et al., 2021). A variety of alternative procedures for the broth micro-dilution have been proposed in recent years, such as modified LN broth FastFung broth and modified Dixon broth (Far et al., 2018; Abdillah et al., 2021). However, there is neither standard parameters including incubation time, culture media and inoculation amount established for the determination of MIC values of *Malassezia* spp. nor susceptibility/resistance breakpoints of antifungal drugs. Thereby, standard procedures for antimicrobial susceptibility profiles of *Malassezia* species needed to be further established.

Azoles, polyenes and echinocandins were the main antifungal agents used to manage various types of fungal infections. The first two antifungal drugs were frequently employed to treat *Malassezia*-related infections in humans and animals. Topical antifungal agents (mainly azoles) were adequate for the treatment of localized skin lesions, while systemic fluconazole (FLZ) or itraconazole (ITZ) for severe skin diseases. Intravenous AmB or its formulations was recommended for the treatment of systemic infections caused by *M. furfur* (Chen et al., 2020; Chen et al., 2021). The antifungal activity of l-AmB was higher than deoxycholate AmB, which might be contributed to the lipophilic nature of this yeast (Iatta et al., 2015). Usually, approximately 24 days of AmB treatment might be useful for a positive outcome of *M. furfur* fungemia (Rhimi et al., 2020). Furthermore, the observed *in vitro* susceptibility values suggested that *Malassezia* might be intrinsically resistant to echinocandins such as caspofungin and micafungin (Rhimi et al., 2021). If echinocandins were used to prevent fungal infection in hospitalized patients, they might be selective for *Malassezia*, potentially leading to *Malassezia*-related infections.

The formation of fungal biofilms not only played an important role in the pathogenesis, but also increased the resistance to immune defense and antifungal therapy (Le Mauff, 2020; Pathakumari et al., 2020). Successful eradication of biofilm-associated fungi was difficult, mainly because it required higher concentrations of antifungal agents than those needed to kill planktonic cells, which were generally toxic to the host. Removal or exchange of medical devices containing biofilms was the most important treatment for biofilm infections (Muakkassa and Ghannoum, 2016). In patients with catheter-related *M. furfur* infections, catheter removal, discontinuation of parenteral lipid nutrition and the institution of systemic antifungal treatment were recommended (Pedrosa et al., 2018).

All-cause mortality rate in this study was 27.9% (24/86). All deaths had CVADs, and 20 patients received TPN infusion. As risk factors for *M. furfur* infection, whether indwelling catheter and TPN infusion were associated with the mortality needed further investigation. Although 17 patients received the recommended AmB or l-AmB treatment, the cause of death was likely related to delayed diagnosis, delayed administration of medication, and failure to combine the removal of the indwelling catheter. However, this was a retrospective study, and the data about the time from hospitalization to diagnosis and the time from diagnosis to medication were missing, so the cause of death could not be further discussed, which was also the limitation of this study.

In summary, we provide a case report and literature review on invasive infections caused by *M. furfur*, demonstrating the epidemiology, risk factors, pathogenesis, diagnosis and treatment. *M. furfur* invasive infections generally present with nonspecific symptoms and occur in preterm infants or patients with immunocompromised or underlying gastrointestinal diseases. Indwelling catheter and TPN infusion are the main risk factors and involved in the pathogenesis of infection. Pathogenic diagnosis requires special attention to the lipophilic characteristics of *M. furfur*. Standard procedures for *in vitro* antimicrobial susceptibility testing of *M. furfur* needed to be further established. Currently, the most commonly used anti-*M. furfur* drugs in clinical practice are azoles and polyenes, especially AmB or l-AMB. Removal of the catheter and termination of TPN infusion are effective in the treatment of *M. furfur* invasive infections.

## Data availability statement

The original contributions presented in the study are included in the article/supplementary material. Further inquiries can be directed to the corresponding authors.

## Ethics statement

The studies involving human participants were reviewed and approved by Ethics Committee of Jiangsu Province Hospital. Written informed consent to participate in this study was provided by the participants’ legal guardian/next of kin. Written informed consent was obtained from the minor(s)’ legal guardian/next of kin for the publication of any potentially identifiable images or data included in this article.

## Author contributions

XZ designed the study, collected the data, and wrote the manuscript. FJ and FN contributed to the fungal identification and manuscript writing. FJ, YX, YL contributed to extract data from literature and drawing of figures and tables in the manuscript. WX and YL contributed to manuscript revisions. All authors contributed to the article and approved the submitted version.
